# Accurate Lens-Distortion Measurement Through Detector Nyquist Sampling

**DOI:** 10.3390/s26051550

**Published:** 2026-03-01

**Authors:** Yongqiang Yang, Zhiyi Wang, Junlin Li, Zhongming Li, Jianlin Lv, Min Zhao, Yanfu Tang, Jianli Wang

**Affiliations:** 1 Changchun Institute of Optics, Fine Mechanics and Physics, Chinese Academy of Sciences, Changchun 130033, China; yangyongqiang@ciomp.ac.cn (Y.Y.); wangzhiyi18@mails.ucas.edu.cn (Z.W.); lijunlin@ciomp.ac.cn (J.L.); lizhongming@ciomp.ac.cn (Z.L.); lvjianlin@ciomp.ac.cn (J.L.); zhaomin@ciomp.ac.cn (M.Z.); tangyanfu@ciomp.ac.cn (Y.T.); 2 University of Chinese Academy of Sciences, Beijing 100049, China

**Keywords:** fringe pattern, Nyquist sampling, phase shift, distortion

## Abstract

**Highlights:**

**What are the main findings?**

- The method enables fast acquisition, high-resolution, and high-precision full-field distortion measurements.
- This method achieved a magnification deviation factor repeatability accuracy of approximately ±108 nm/cm and third-order distortion-measurement accuracy of approximately ±108 nm/cm^3^.

**What are the implications of the main findings?**

- This approach enables a high-precision distortion evaluation for conventional industrial imaging lenses.
- The derived imaging-magnification factor facilitates a more accurate determination of the focal-plane position of the lens.

**Abstract:**

Distortion is a key parameter affecting the imaging performance of lenses. In this study, we propose a testing method based on detector Nyquist sampling of image data to achieve high-precision measurements of the distortion distribution of lenses. The distribution patterns of distortions in horizontal and vertical directions can be obtained by analyzing the distribution patterns of Moiré fringes in images under Nyquist sampling conditions and using phase-shift algorithms. The distortion-distribution characteristics of the lens are then calculated using distortion formulas. This method is characterized by high testing accuracy and sampling resolution. The image-plane distortion distribution exhibited a consistent linear trend when the object-plane position varied within a limited spatial range. Furthermore, the proposed method achieved a magnification deviation factor repeatability accuracy of approximately ±108 nm/cm and third-order distortion-measurement accuracy of approximately ±108 nm/cm^3^. This method enables a high-precision distortion evaluation of conventional industrial imaging lenses.

## 1. Introduction

Lens distortion is a critical factor affecting image accuracy in modern optical systems, particularly high-precision applications such as semiconductor lithography, aerial imaging, and machine vision [1]. Distortion refers to the deviation of actual image points from their ideal positions and is primarily caused by optical aberrations, mechanical assembly errors, and environmental factors.

Distortion can lead to the inaccurate determination of the geometric positions and dimensions of objects in images and may even result in misjudgments during image-recognition processes [2]. Therefore, measuring the distortion in optical systems is crucial for any technology that involves computer-vision tasks. Distortion-testing methods primarily include template-based measurements, multi-field-of-view approaches, and plumb-line techniques. Template-based methods utilize known reference patterns such as checkerboards or feature-point configurations to determine the distortion parameters for image correction [3,4,5,6,7,8,9,10]. However, these methods are highly sensitive to environmental factors such as lighting conditions and the placement of the calibration board. Multi-field-of-view approaches estimate distortion parameters by analyzing point correspondences in distorted images captured from different perspectives, including rotation-based methods and modulation transfer function (MTF)-based techniques [11,12,13]. However, these methods require specialized equipment and expertise for testing and analysis. Plumb-line methods detect distortion parameters based on the characteristic of straight lines becoming distorted into arcs [14,15,16]. This approach enables corrections using only a single distorted image; however, it depends on the presence of a sufficient number of straight-line structures within the original field of view. Furthermore, the presence of ambiguous or nondistorted arcs in an image may result in detection errors that typically require manual intervention.

Fringe phase-analysis technology, known for its high-precision and automated full-field analysis capabilities, is widely utilized in various optical fringe-based measurement techniques, such as interferometry, digital holography, Moiré-fringe measurements, and fringe-projection profilometry. Researchers have also applied this method to the measurement of lens distortion by employing techniques such as carrier-fringe phase analysis and fringe phase-shifting analysis. The carrier-fringe phase analysis utilizes Fourier transform-based methods to analyze carrier fringes in lens-distortion testing [17]. However, Fourier transform is a global operation; therefore, detecting and extracting the fundamental frequency of carrier fringes can introduce significant uncertainties, which may compromise the accuracy of the correction results. Fringe phase-shifting analysis involves multistep phase shifting to demodulate the phase and obtain the phase distribution of distorted fringe images [18,19,20]. This method provides a high phase-calculation accuracy, enabling precise distortion evaluation. However, the accuracy of phase-shifting calculations is influenced by the fringe period. Under the same resolution conditions, smaller fringe periods result in a higher distortion-calculation accuracy. However, denser fringes within a unit length may lead to insufficient sampling because of the limited resolution of the detector, thereby affecting the accuracy of the phase-shifting calculations.

In this study, we investigated the phase-distribution characteristics of fringe images under the Nyquist sampling condition of detectors and explored a multistep phase-shifting calculation process based on contrast-based methods. We improved the algorithm to develop a phase-shifting processing technique adaptable to various testing conditions. In high-precision finite-distance imaging optical systems, the proposed approach enables accurate measurement of lens distortion and enhances the precision of image recognition and positioning. In addition, the imaging-magnification factor derived during the computation process facilitates a more accurate determination of the focal-plane position of the lens.

## 2. Principle

The mathematical characterization of lens distortion primarily involves three widely recognized models: Brown’s even-order polynomial formulation, Fitzgibbon’s division-based model [21], and the parametric-distortion model commonly used in lithographic applications, which incorporates translation, rotation, magnification, and third-order distortion components [22]. In our experimental methodology, the spatial configuration of the fringe-test target inherently introduced rotational and magnification artifacts during the measurement process. These systematic errors must be computationally decoupled during the extraction of the distortion parameters. The mathematical formulation of the distortion model is as follows:

(1) $$dx=T_{x}+\Delta M_{x}x-\theta_{x}y+D_{3x}x(x^{2}+y^{2}),$$

(2) $$dy=T_{y}+\Delta M_{y}y+\theta_{y}x+D_{3y}y(x^{2}+y^{2}),$$

where dx and dy represent positional deviations between the ideal and actual image points, expressed in pixels and micrometers, respectively. $T_{x}$ and $T_{y}$ denote the optical-center offset. In most cases, the optical center of the lens is used as the reference point for distortion, which results in near-zero offset values in well-aligned systems. $\theta_{x}$ and $\theta_{y}$ correspond to angular-displacement components. When the direction of the striped target is not aligned with the orientation of the detector pixel array, the angular terms introduce the corresponding components into the phase distribution. $\Delta M_{x}$ and $\Delta M_{y}$ are magnification coefficients. Any deviation in the actual placement of the target from its intended position leads to a discrepancy between the captured fringe pattern and designed optical magnification. $D_{3x}$ and $D_{3y}$ represent the third-order radial-distortion components along the *x-* and *y*-axes, respectively. Higher-order distortion terms (fifth order and higher) were omitted from this model because their magnitudes were generally below the threshold of practical significance [23]. The most appropriate model may also be selected based on the lens’s specific characteristics.

The parameters described above were determined by applying a least-squares fitting method to the displacement matrices (dx and dy) obtained through a multistep phase-shifting analysis. The corresponding computational formula is as follows:

(3) $$\left[\begin{array}{c} dx_{1}\\ dy_{1}\\ dx_{2}\\ dy_{2}\\ \vdots \\ dx_{n}\\ dy_{n} \end{array}\right]=\left[\begin{array}{cccccccc} 1 & 0 & x_{1} & 0 & -y_{1} & 0 & x_{1}(x_{1}^{2}+y_{1}^{2}) & 0\\ 0 & 1 & 0 & y_{1} & 0 & x_{1} & 0 & y_{1}(x_{1}^{2}+y_{1}^{2})\\ 1 & 0 & x_{2} & 0 & -y_{2} & 0 & x_{2}(x_{2}^{2}+y_{2}^{2}) & 0\\ 0 & 1 & 0 & y_{2} & 0 & x_{2} & 0 & y_{2}(x_{2}^{2}+y_{2}^{2})\\ \vdots & \vdots & \vdots & \vdots & \vdots & \vdots & \vdots & \vdots \\ 1 & 0 & x_{n} & 0 & -y_{n} & 0 & x_{n}(x_{n}^{2}+y_{n}^{2}) & 0\\ 0 & 1 & 0 & y_{n} & 0 & x_{n} & 0 & y_{n}(x_{n}^{2}+y_{n}^{2}) \end{array}\right]\left[\begin{array}{c} T_{x}\\ T_{y}\\ \Delta M_{x}\\ \Delta M_{y}\\ \theta_{x}\\ \theta_{y}\\ D_{3x}\\ D_{3y} \end{array}\right].$$

**Figure 1:**
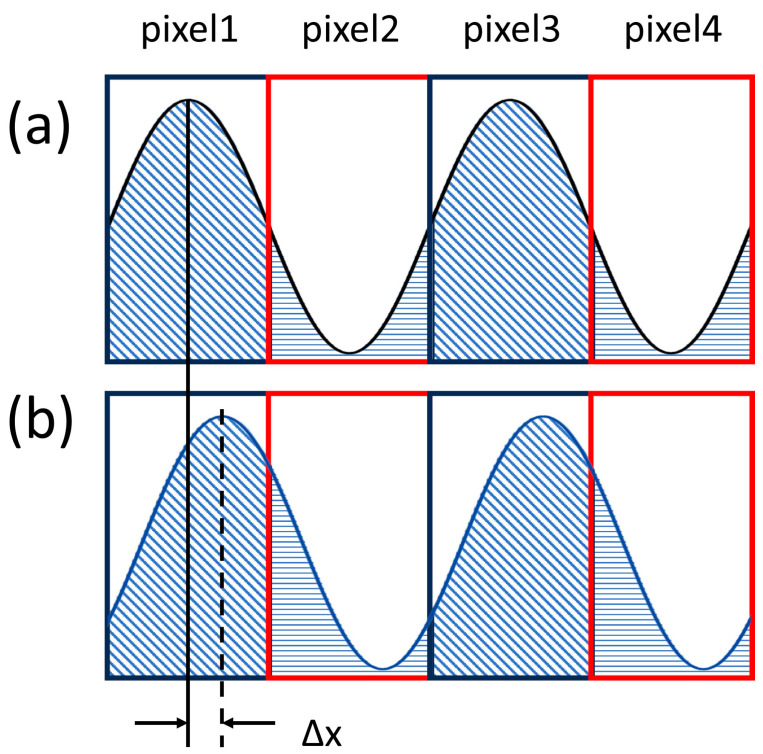
Schematic of fringe-energy integration process. (**a**) Positional relationship between the fringe with an initial phase of 0° and pixel elements under distortion-free conditions. (**b**) Positional relationship between the fringe with an initial phase of 0° and pixel elements under distorted conditions.

At the Nyquist frequency, the sampling process by the detector involves integrating the high- and low-intensity regions of the fringe pattern through adjacent pixels [24]. This integration yields alternating bright and dark fringe patterns, as shown in Figure 1a. When the fringe pattern is ideally focused onto the detector through the lens, the detection surface exhibits periodic patterns of bright and dark fringes. Deviations in the fringe positions arise when the lens introduces distortions or when discrepancies exist in the magnification factor, as illustrated in Figure 1b. The magnitude of the deviation between the ideal and actual positions of the fringes is phase dependent. By expressing the fringe distribution using a sine function, the relationship between the deviation and phase can be described as follows:

(4) $$I=\sin(2\pi f_{0}a+\varphi ),$$

(5) $$dx=\frac{\varphi }{2\pi f_{0}}=\frac{\varphi }{2\pi }l_{fri}.$$

The coefficient $f_{0}$ is the spatial frequency corresponding to this period, and $l_{fri}$ denotes the length of a single period of the fringe pattern. In the ideal state, $l_{fri}$ is equivalent to two pixels. When a shift occurs, the fringe pattern undergoes displacement dx. The corresponding calculation for φ is shown in Equation (5). Achieving a sinusoidal energy distribution in the actual testing processes is challenging. Therefore, rectangular fringe targets are typically used for testing.

**Figure 2:**
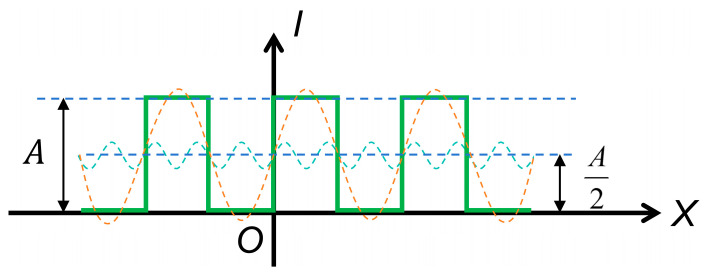
Intensity distribution curve of a rectangular fringe target. The solid green line represents the rectangular fringe; the orange dashed line represents the fundamental frequency component; and the cyan dashed line represents the higher-order frequency component.

As shown in Figure 2, the Fourier transform of a rectangular fringe target includes multiple frequency components that affect the phase-shifting process, thereby necessitating further analysis. The light intensity is expressed as follows:

(6) $$I(x)=\frac{A}{2}+\frac{2A}{\pi }\sum_{n=1}^{\infty }\frac{\sin(2\pi (2n-1)f_{0}x+(2n-1)\varphi (x))}{2n-1}.$$

After passing through the imaging system, each frequency component is modulated using the optical transfer function (OTF). The OTF is typically expressed as $OTF(f)=MTF(f)e^{iPTF(f)}$, where MTF(f) and PTF(f) are the modulation and phase transfer functions. To simplify the analysis, the phase distortion introduced by the phase transfer function at the frequency $(2n-1)f_{0}$ is assumed to be (2n−1)φ(x), which may vary with the spatial position *x*. Then, the output intensity signal is given by

(7) $$I^{\prime }(x)=\frac{A}{2}+\frac{2A}{\pi }\sum_{n=1}^{\infty }MTF((2n-1)f_{0})\frac{\sin(2\pi (2n-1)f_{0}x+(2n-1)\varphi (x))}{2n-1}.$$

The average intensities corresponding to the bright and dark stripes of the square-wave target must be computed to calculate the contrast transfer function (CTF). Let one period of the square-wave target be $1/f_{0}$, with the bright stripe interval $\left[a,a+1/(2f_{0})\right]$ and dark stripe interval $\left[a+1/(2f_{0}),a+1/f_{0}\right]$, where a is the initial phase. The average intensities are as follows:

(8) $$I_{\max}=\int_{a}^{a+\frac{1}{2f_{0}}}I^{\prime }(x)dx=\frac{A}{4f_{0}}+\frac{2A}{\pi }\sum_{n=1}^{\infty }MTF((2n-1)f_{0})\frac{J_{2n-1}^{+}}{2n-1},$$

(9) $$I_{\min}=\int_{a+\frac{1}{2f_{0}}}^{a+\frac{1}{f_{0}}}I^{\prime }(x)dx=\frac{A}{4f_{0}}+\frac{2A}{\pi }\sum_{n=1}^{\infty }MTF((2n-1)f_{0})\frac{J_{2n-1}^{-}}{2n-1},$$

where the integral terms are

(10) $$J_{2n-1}^{+}=\int_{a}^{a+\frac{1}{2f_{0}}}\sin(2\pi (2n-1)f_{0}x+(2n-1)\varphi (x))dx,$$

(11) $$J_{2n-1}^{-}=\int_{a+\frac{1}{2f_{0}}}^{a+\frac{1}{f_{0}}}\sin(2\pi (2n-1)f_{0}x+(2n-1)\varphi (x))dx.$$

The CTF is defined as the normalized difference between the average intensities of bright and dark stripes:

(12) $$CTF=\frac{I_{\max}-I_{\min}}{I_{\max}+I_{\min}}=\frac{\frac{2A}{\pi }\sum_{n=1}^{\infty }\frac{MTF((2n-1)f_{0})}{2n-1}\left(J_{2n-1}^{+}-J_{2n-1}^{-}\right)}{\frac{A}{2f_{0}}+\frac{2A}{\pi }\sum_{n=1}^{\infty }\frac{MTF((2n-1)f_{0})}{2n-1}\left(J_{2n-1}^{+}+J_{2n-1}^{-}\right)}.$$

As φ(x) is a function of x, the integrals $J_{2n-1}^{\pm }$ generally have no analytical solution. If the phase distortion varies slowly within half a period, then it can be approximated by its value at the midpoint of each interval. Let

(13) $$\varphi_{1}\approx \varphi \left(a+\frac{1}{4f_{0}}\right),$$

(14) $$\varphi_{2}\approx \varphi \left(a+\frac{3}{4f_{0}}\right).$$

Then, the integrals can be approximated as

(15) $$J_{2n-1}^{+}\approx \frac{\cos(2\pi (2n-1)f_{0}a+(2n-1)\varphi_{1})}{\pi (2n-1)f_{0}},$$

(16) $$J_{2n-1}^{-}\approx -\frac{\cos(2\pi (2n-1)f_{0}a+(2n-1)\varphi_{2})}{\pi (2n-1)f_{0}}.$$

Substituting Equations (15) and (16) into Equations (8) and (9) and then into Equation (12) yields the following:

(17) $$CTF\approx \frac{\frac{4}{\pi^{2}}\sum_{n=1}^{\infty }\frac{MTF((2n-1)f_{0})}{{\left(2n-1\right)}^{2}}\left(\cos(2\pi (2n-1)f_{0}a+(2n-1)\varphi_{1})+\cos(2\pi (2n-1)f_{0}a+(2n-1)\varphi_{2})\right)}{1+\frac{4}{\pi^{2}}\sum_{n=1}^{\infty }\frac{MTF((2n-1)f_{0})}{{\left(2n-1\right)}^{2}}\left(\cos(2\pi (2n-1)f_{0}a+(2n-1)\varphi_{1})-\cos(2\pi (2n-1)f_{0}a+(2n-1)\varphi_{2})\right)}.$$

Image sensors typically perform the equidistant sampling of a spatial signal. In the calculation process of the CTF, the size of a corresponds to twice the pixel size, and the relationship between a and x is as follows:

(18) $$a_{k}=x_{2k}=p_{0}+2k\Delta ,k\in \mathbb{Z}.$$

The pixel size of an image sensor is typically at the micrometer level. Within a small range, the distortion of the optical system can be considered approximately equal, that is, $\varphi_{1}\approx \varphi_{2}\approx \varphi (x_{2k})$. Substituting this into Equation (17) yields the following:

(19) $$CTF\approx \frac{8}{\pi^{2}}\sum_{n=1}^{\infty }\frac{MTF((2n-1)f_{0})}{{\left(2n-1\right)}^{2}}\cos(2\pi (2n-1)f_{0}a_{k}+(2n-1)\varphi (x_{2k})).$$

As shown in Equations (8) and (9), the grayscale distribution of the rectangular fringe patterns after pixel integration retains higher-order harmonics, such as the third and fifth harmonics of the Nyquist frequency. The CTF analysis based on Equation (19) indicates that the contrast of the fringes is mainly determined by the MTF of the lens.

To analyze the influence of higher-order harmonics on the phase-shifting process, Equation (19) is simplified. Considering that most optical lenses effectively attenuate frequencies above the seventh order, the following analysis only accounts for the effects of 3rd and 5th harmonics. The new intensity distribution obtained after the CTF calculation is shown in Equation (20).

(20) $$I(x)=\frac{8}{\pi^{2}}\sum_{m=1,3,5}\frac{MTF(mf_{0})}{m^{2}}\cos(2\pi mf_{0}x+m\varphi ).$$

In this study, the four-step phase-shift method, which is a classical phase-shift algorithm, is analyzed to evaluate the impact of higher-order harmonics on the measurement accuracy. Using Equation (21) and by shifting the target by specific distances along the *x*-direction to achieve shifts phases of 0, π/2, π, and 3π/2, we can determine the intensity distribution for the four-step phase-shift, as shown in Equations (22)–(25):

(21) $$I_{k}=\frac{8}{\pi^{2}}\sum_{m=1,3,5}\frac{MTF(mf_{0})}{m^{2}}\cos(m\delta_{k}+m\varphi ).$$

(22) $$I_{1}=\frac{8}{\pi^{2}}\left(MTF(f_{0})\cos(\varphi )+\frac{MTF(3f_{0})}{9}\cos(3\varphi )+\frac{MTF(5f_{0})}{25}\cos(5\varphi )\right).$$

(23) $$I_{2}=\frac{8}{\pi^{2}}\left(-MTF(f_{0})\sin(\varphi )+\frac{MTF(3f_{0})}{9}\sin(3\varphi )-\frac{MTF(5f_{0})}{25}\sin(5\varphi )\right).$$

(24) $$I_{3}=\frac{8}{\pi^{2}}\left(-MTF(f_{0})\cos(\varphi )-\frac{MTF(3f_{0})}{9}\cos(3\varphi )-\frac{MTF(5f_{0})}{25}\cos(5\varphi )\right).$$

(25) $$I_{4}=\frac{8}{\pi^{2}}\left(MTF(f_{0})\sin(\varphi )-\frac{MTF(3f_{0})}{9}\sin(3\varphi )+\frac{MTF(5f_{0})}{25}\sin(5\varphi )\right).$$

The relationship between phase and energy is expressed in the form of a tangent function:

(26) $$\varphi_{calc}=\arctan\left(\frac{I_{4}-I_{2}}{I_{1}-I_{3}}\right).$$

Substituting Equations (22)–(25) into this expression yields Equation (26), where $r_{3}=\frac{MTF(3f_{0})}{9MTF(f_{0})}$, $r_{5}=\frac{MTF(5f_{0})}{25MTF(f_{0})}$:

(27) $$\tan\varphi_{calc}=\frac{I_{4}-I_{2}}{I_{1}-I_{3}}=\frac{\sin\varphi -r_{3}\sin3\varphi +r_{5}\sin5\varphi }{\cos\varphi +r_{3}\cos3\varphi +r_{5}\cos5\varphi }.$$

We assume that the true and calculated phases are denoted by φ and $\varphi_{calc}=\varphi +\Delta \varphi$, (where Δφ is a small quantity), respectively. Then, using Taylor expansion, we obtain

(28) $$\tan(\varphi +\Delta \varphi )\approx \tan\varphi +(1+\tan^{2}\varphi )\Delta \varphi =\tan\varphi +\frac{\Delta \varphi }{\cos^{2}\varphi }.$$

Simultaneously, we expand the expression $\tan\varphi_{calc}$ under the condition that $r_{3}$ and $r_{5}$ are small quantities:

(29) $$\begin{array}{l} \tan\varphi_{calc}\approx \frac{\sin\varphi }{\cos\varphi }+\frac{(-r_{3}\sin3\varphi +r_{5}\sin5\varphi )\cos\varphi -(r_{3}\cos3\varphi +r_{5}\cos5\varphi )\sin\varphi }{\cos^{2}\varphi }\\ \;\;\;\;\;=\tan\varphi +\frac{-r_{3}(\sin3\varphi \cos\varphi +\cos3\varphi \sin\varphi )+r_{5}(\sin5\varphi \cos\varphi -\cos5\varphi \sin\varphi )}{\cos^{2}\varphi }\\ \;\;\;\;\;=\tan\varphi +\frac{(r_{5}-r_{3})\sin4\varphi }{\cos^{2}\varphi } \end{array}.$$

A comparison of Equations (28) and (29) yields

(30) $$\Delta \varphi \approx (r_{5}-r_{3})\sin4\varphi .$$

According to Equation (30), the phase error varies with the phase, and its magnitude is influenced by the ratio of higher-order harmonics to the first-order harmonic. A small ratio results in a small phase error. Typically, as the frequency increases, the MTF of higher-order frequencies declines rapidly. Moreover, the coefficient $1/n^{2}$, associated with the harmonic order in the formula, further reduces the phase error introduced by higher-order harmonics. Depending on the MTF characteristics of the lens under test in different measurement scenarios, multistep phase-shifting algorithms (such as five-step or eight-step phase shifting) can be selected to effectively suppress the influence of harmonics, such as the third- and fifth-order harmonics.

## 3. Method

**Figure 3:**
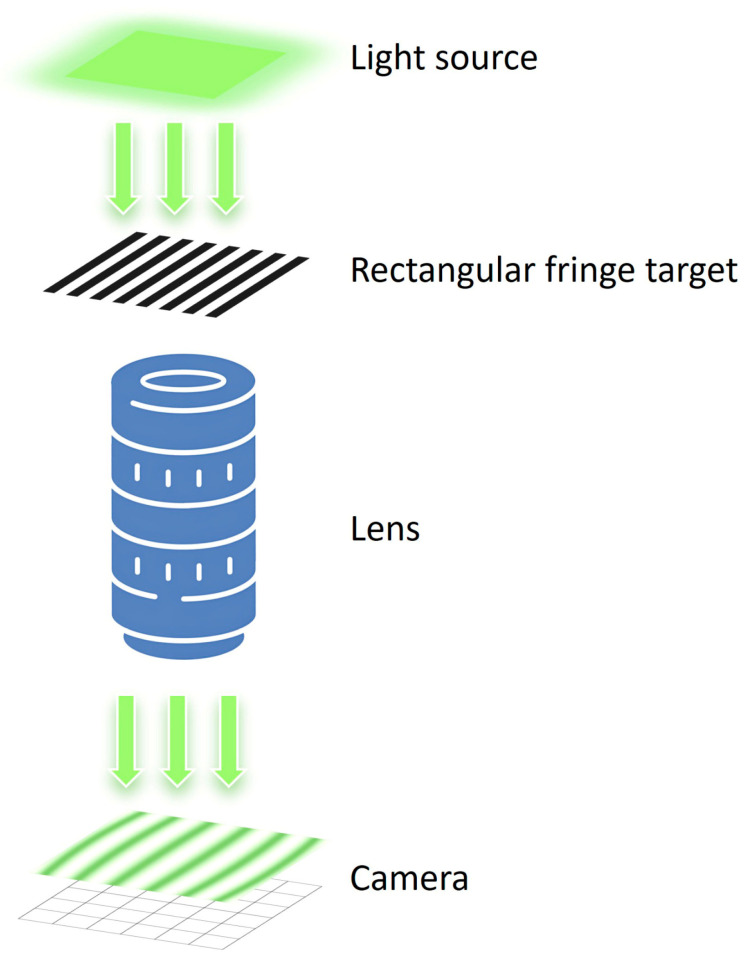
Schematic of the test-setup configuration.

The experimental setup illustrated in Figure 3 was employed to characterize the distortion distribution across the entire field of view. A rectangular fringe target was positioned on the object plane of the lens, and its dimensions were specifically designed to fully cover the imaging area. The detector was placed in the image plane to capture fringe patterns under uniform illumination. During the experiment, the rectangular fringe target could perform phase-shifting movements along both the *x*- and *y*-directions. Moreover, both the fringe target and detector could be adjusted along the *z*-axis to achieve optimal focus and precise control of magnification. Owing to the one-dimensional nature of the fringe pattern, the target had to be rotated by exactly 90° to enable phase-shifting measurements along the orthogonal directions. The following derivation assumes the ideal Nyquist-sampling condition (2 pixels per fringe period) and a corresponding phase-shift step of π/2 radians between intensity acquisitions for the contrast calculation.

**Figure 4:**
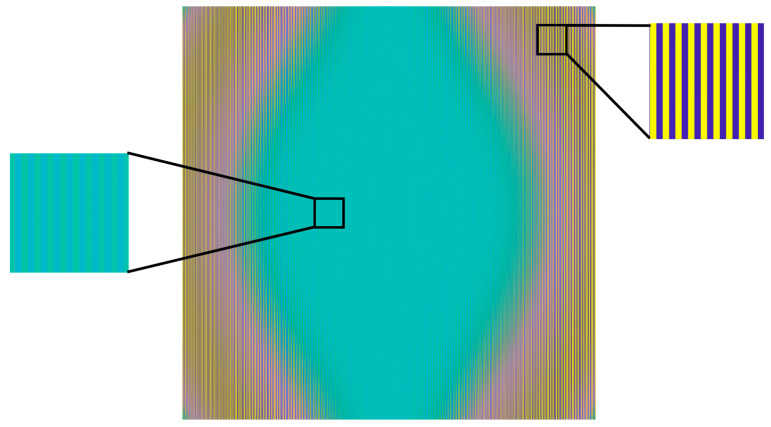
Effect of distortion distribution on fringe patterns in the Nyquist sampling process.

A simulation-based approach was employed to intuitively analyze the influence of distortion on the imaging process of rectangular fringe targets. Assuming no magnification deviation, a third-order distortion-distribution matrix was generated based on Equation (1). This distortion model was then applied to the sinusoidal fringes using Equations (31) and (32). The resulting distorted fringes were sampled at the Nyquist frequency, and the corresponding imaging outcomes are shown in Figure 4.

(31) $$I_{x}=\sin(2\pi f_{0}x+\varphi_{x}(x,y)),$$

(32) $$I_{y}=\sin(2\pi f_{0}y+\varphi_{x}(x,y)).$$

**Figure 5:**
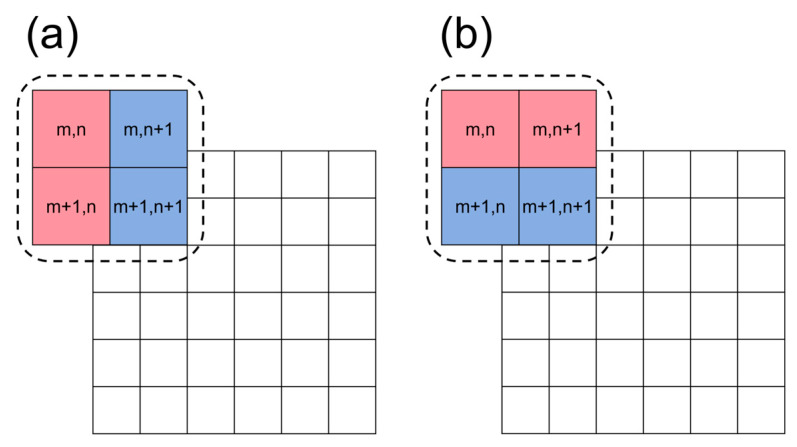
Schematic of contrast processing. (**a**) Horizontal-direction contrast processing. (**b**) Vertical-direction contrast processing.

**Figure 6:**
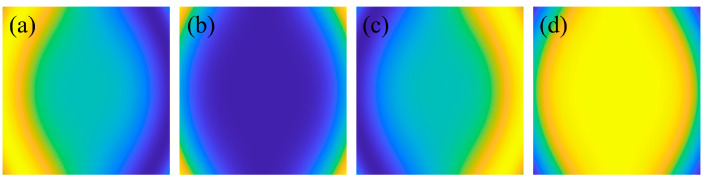
Intensity-variation distribution in four-step phase-shifting process. (**a**) First step phase-shifted image. (**b**) Second step phase-shifted image. (**c**) Third step phase-shifted image. (**d**) Fourth step phase-shifted image.

Evidently, the distortion was relatively minor in the central region of the detector. When two adjacent pixels in the center sample the bright and dark fringe centers, low-contrast Moiré fringes are generated, as illustrated by the magnified central portion on the left side of Figure 4. In regions farther from the center, as the distortion increases, the pixel sampling of the sinusoidal signal introduces phase shifts, causing variations in the grayscale values of adjacent pixels, thereby altering the contrast. This effect is demonstrated in the magnified image in the upper-right corner of Figure 4. This figure clearly illustrates that the distribution of distortion influences the shape of the Moiré fringes. Images containing Moiré fringes require pre-processing before phase shifting to address the issue of the insufficient sampling of periodic signals. As derived in Equation (19), performing contrast calculations on the images reveals that the resulting data contain phase information influenced by distortion. The procedure for the contrast calculation is illustrated in Figure 5. For the phase shift in both the *x*- and *y*-directions, contrast calculations can be applied to the four adjacent pixels using Equations (33) and (34). This metric is defined as the CTF, which is widely employed in optical and imaging system analysis and is correlated to the more common MTF [25]. Notably, each pixel should participate in the contrast calculation only once; otherwise, the influence of bright and dark fringes cannot be eliminated, leading to an abnormal Moiré-fringe extraction. After the contrast calculation, the image resolution perpendicular to the fringe direction was reduced to half of the original resolution. To maintain the same aspect ratio between the horizontal and vertical resolutions, the minimum calculation unit consisted of four adjacent pixels. The simulated four-step phase-shifting patterns are shown in Figure 6. As the phase varied, the distribution of the Moiré fringes changed accordingly.

(33) $$CTF_{x}(m,n)=\frac{\left(I(m,n)+I(m+1,n))-(I(m,n+1)+I(m+1,n+1)\right)}{\left(I(m,n)+I(m+1,n))+(I(m,n+1)+I(m+1,n+1)\right)},$$

(34) $$CTF_{y}(m,n)=\frac{\left(I(m,n)+I(m,n+1))-(I(m+1,n)+I(m+1,n+1)\right)}{\left(I(m,n)+I(m,n+1))+(I(m+1,n)+I(m+1,n+1)\right)}.$$

**Figure 7:**
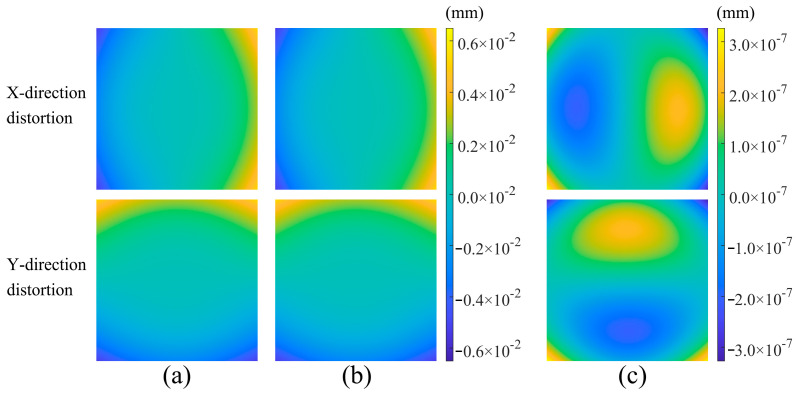
Simulation results of distortion calculation. (**a**) Simulated input values for *x*- and *y*-direction distortions. (**b**) Distortion-calculation results in *x*- and *y*-directions. (**c**) Deviation between calculated and input values in *x*- and *y*-directions.

The four-step phase-shifting method was employed to process and unwrap the phase-shifting patterns in both *x*- and *y*-directions. As illustrated in Figure 7, the eight-bit phase-shifting images produced a calculated distortion distribution whose deviation from the preset distortion values was considerably smaller than the set value. This indicates that the method possessed a high-precision capability for assessing the distortion distribution of optical lenses.

## 4. Experimental Setup

**Figure 8:**
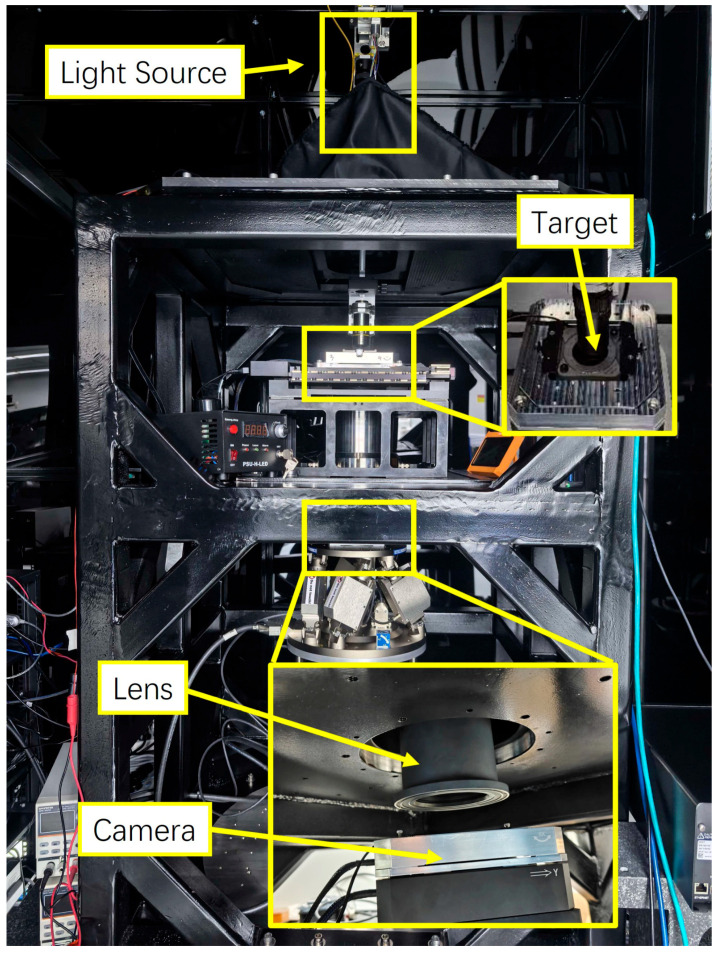
Photograph of the experimental test setup.

The distortion testing of the self-developed optical lens was performed using the experimental setup shown in Figure 8. The lens was designed with a 1:1 magnification ratio, an MTF cutoff frequency of approximately 100 lp/mm, and a numerical aperture of 0.24. During the optical design and simulation process, compensation was achieved through a combination of image-plane translation and adjustment of the post-stop spacing; consequently, the maximum variation in the chief ray height could be controlled to within 1.9 μm. Combined with the alignment simulation analysis, the predicted maximum value of the actual distortion distribution was less than 10 μm. The image-sensor model used in the test was a MARS-3250-12U3MC (Daheng Imaging, Beijing, China) with a pixel size of 3.2 μm. The sensor was operated in the 4 × 4 binning mode (effective pixel size 12.8 μm) to ensure that the imaged fringe pattern appeared at a spatial frequency where the lens under test provided a sufficiently high MTF (MTF > 0.2). This operation condition guaranteed high-contrast signals for precise phase-shift analysis. The central rectangular region was extracted for computation at a resolution of 1234 × 1234 pixels.

A one-dimensional rectangular fringe mask (STARMASK, ShenZhen, China), with a period of 25.6 μm, was designed based on the lens-magnification ratio and target spatial frequency. The image sensor had an active imaging area of 15.7952 mm × 15.7952 mm. The fringe mask was fabricated using the laser direct-writing technology with a feature accuracy of 50 nm, corresponding to a maximum position deviation of ±25 nm, which ensured the accuracy of the measurements. The pattern diameter was 30 mm, which was sufficient to adequately cover the test area. Precision-manufactured sensor pixels effectively provided an ideal detection surface for the experiment. The mask was rotated by the rotation stage (Custom-made, Atto Motion Instruments, Rizhao, China), which featured a repetitive positioning accuracy better than 20 arcseconds. The 0° and 90° orientations were precisely calibrated once prior to the measurements, and the high repeatability of the stage ensured consistent alignment. Simulation analyses confirmed that compared to the measurement repeatability, the angular errors within this range only negligibly influenced the calculated distortion parameters.

The translation stages (P15.XY100S-C, COREMORROW, Harbin, China) carrying the rectangular fringe mask utilize piezoelectric-driven mechanical structures to enhance phase-shifting accuracy. The angular deviation between the rectangular target and horizontal plane of the lens flange is less than 30 arcseconds. The image-sensor mounting mechanism employs a hexapod displacement platform (H-824.G2, Physik Instrumente, Karlsruhe, Germany) to precisely align the sensor with the fringe-pattern image, thereby minimizing defocusing-induced measurement errors. The experimental setup was placed on a marble vibration isolation platform to reduce the impact of vibration on the measurements. An integrating sphere, combined with a light-emitting diode (LED) light source, was used for uniform illumination. The LED had a central wavelength of 530 nm and a full width at half maximum of 20 nm, which matched the designed spectral range of the test optical lens. During testing, the maximum digital number value of the acquired grayscale images was maintained below 220 to prevent saturation from affecting measurement accuracy.

**Figure 9:**
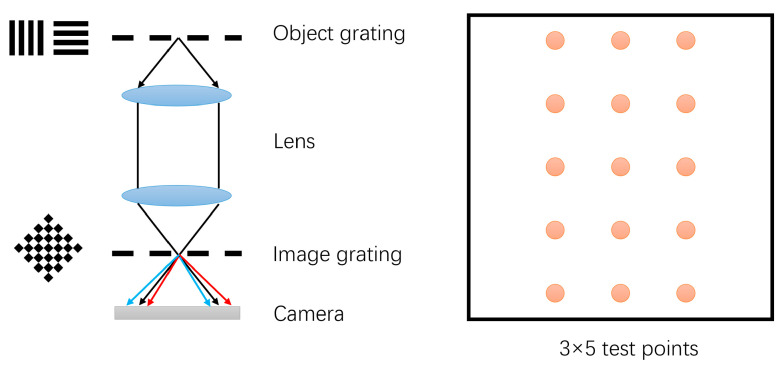
Schematic of the Ronchi testing method. The setup consists of an object grating, a lens under test (LUT), an image grating, and a camera. The right panel illustrates the distribution of the 3×5 test points within the field of view.

To evaluate the measurement accuracy of this method, a Ronchi-based test setup was incorporated into in the experimental apparatus by replacing the object- and image-plane gratings (Figure 9). Simultaneous testing across multiple fields of view could be achieved by fabricating multiple field-of-view mask patterns on both the object and image planes. The Ronchi testing method measured wavefront aberrations in different fields of view and calculated the distortion distribution using the Z2 and Z3 coefficients derived from the test results. The Ronchi shearing interferometry is extensively adopted in lithography lens qualification. Owing to its explicitly defined theoretical model and traceable error sources, its measurement uncertainty can be rigorously characterized. Consequently, the Ronchi test results serve as statistically credible ground truths for validating the accuracy of the proposed method [26].

To rigorously validate the reliability of the reference benchmark, the measurement uncertainty of the Ronchi setup was evaluated following the ISO Guide to the Expression of Uncertainty in Measurement (GUM). The evaluation incorporated both Type A (derived from statistical repeatability) and Type B uncertainties (including mark positioning accuracy, vibration, and phase-shifting errors). The detailed uncertainty budget is summarized in Table 1.

Furthermore, a Monte Carlo simulation was performed to propagate the combined standard uncertainty of the Ronchi method to the lens parameters. The simulation results indicated standard uncertainties of approximately 33 nm/cm and 67 nm/cm^3^ for magnification and third-order distortion, respectively. These values confirm that the Ronchi test possesses sufficient precision to serve as a valid ground truth for evaluating the accuracy of the proposed Nyquist-sampling method.

**Figure 10:**
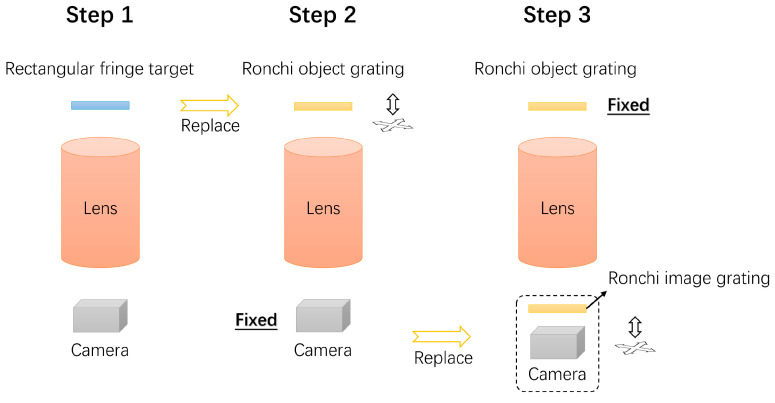
Assembly and alignment flowchart.

To ensure the high precision of the Ronchi test and the validity of the comparison, a rigorous three-step alignment procedure was implemented (Figure 10).

Step 1: The system was initially configured using the rectangular fringe target for the Nyquist sampling test.

Step 2 (Object Alignment): The rectangular target was replaced by the Ronchi object grating, whereas the camera was strictly fixed to maintain the image-plane reference. Then, the object grating was meticulously adjusted to align with the optical axis and minimize wavefront tilt.

Step 3 (Image Alignment): With the fixed object grating, the standard camera was replaced by the Ronchi shearing detection unit (integrating the image grating and sensor). This unit was adjusted to generate null fringes, ensuring conjugacy with the object grating.

The primary objective of this rigorous alignment process was to guarantee the spatial consistency of the measured fields between the two methods. Because lens distortion is inherently field-dependent, precise one-to-one correspondence of the field points (field of view) is a prerequisite for a valid comparison. By fixing the lens assembly as a common datum and strictly aligning the Ronchi gratings to the same optical axis as that of the Nyquist target, we minimize any lateral or rotational spatial mismatch. This approach ensures that both methods characterize the wavefront aberrations at identical physical locations within the lens’s field of view, thereby eliminating errors induced by field coordinate deviations.

## 5. Results and Discussion

**Figure 11:**
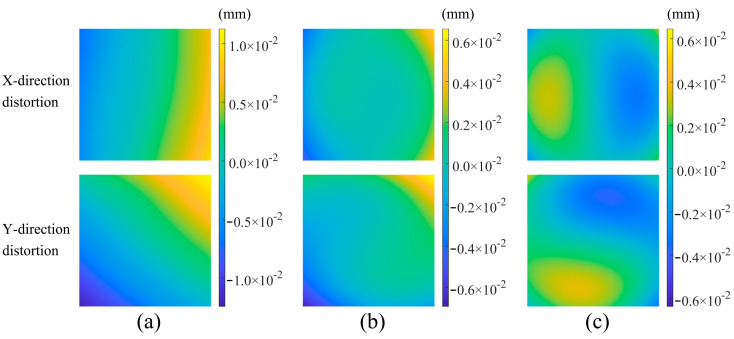
Distortion test results during target imaging at various object-plane positions. (**a**) X- and Y-direction calculation at an object-plane position of −2 mm. (**b**) X- and Y-direction calculation at an object-plane position of 0 mm. (**c**) X- and Y-distortion calculation at an object-plane position of +2 mm.

The aforementioned motion mechanism and rectangular fringe target were employed to test the optical lens. Figure 11 displays the phase-shifting calculation results at object-plane positions of −2, 0, and 2 mm, with 0 mm representing the theoretically designed object plane. Through precise structural design, a rectangular fringe target was positioned at this location, and adjustments to the object-plane position were achieved by varying the height of the spacer blocks. Subsequently, the detector was used to capture images of the rectangular fringes in the image plane of the lens.

**Figure 12:**
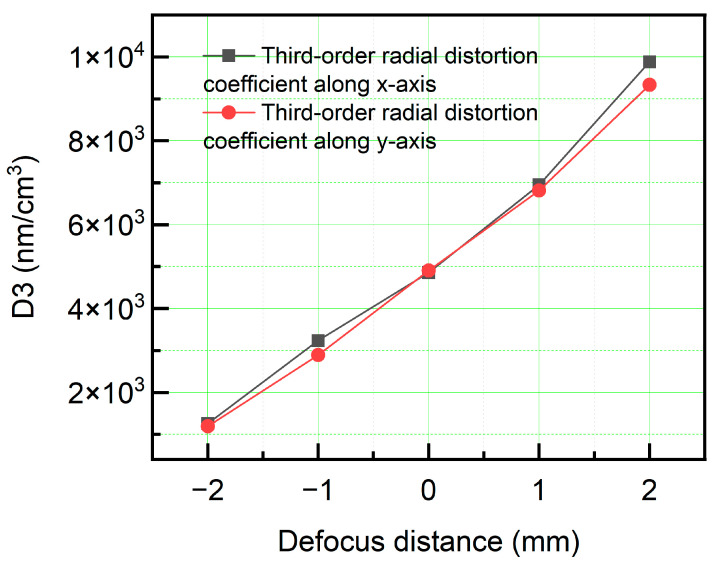
Distribution of third-order distortion coefficients across object-plane positions.

**Figure 13:**
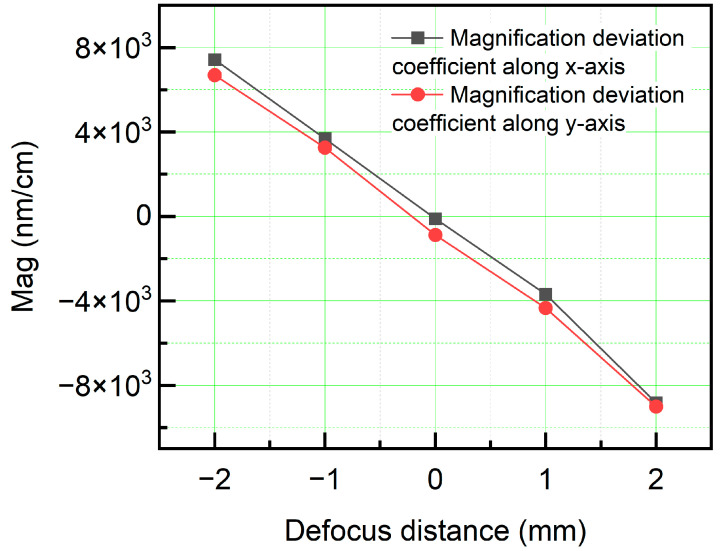
Distribution curves of magnification factor across object-plane positions.

As observed in the figure, the distortion distributions in both the *x*- and *y*-directions changed with variations in the focal-plane position. Given that the lens adopted a double-telecentric design, the overall distortion remained minimal. To achieve a more accurate analysis of the distortion-distribution characteristics, Equation (3) was employed to compute the distortion-distribution pattern, as shown in Figure 12. As the focal-plane position increased, the third-order distortion coefficients in both the *x*- and *y*-directions exhibited a continuous upward trend. Furthermore, the magnification deviation coefficient was evaluated, as shown in Figure 13. The magnification gradually decreased with an increase in the focal-plane position, which is consistent with the fundamental imaging principle. When the object distance increased, the resulting image size decreased. Owing to the limited range of displacement, the magnification deviation coefficient exhibited a relatively linear behavior.

**Figure 14:**
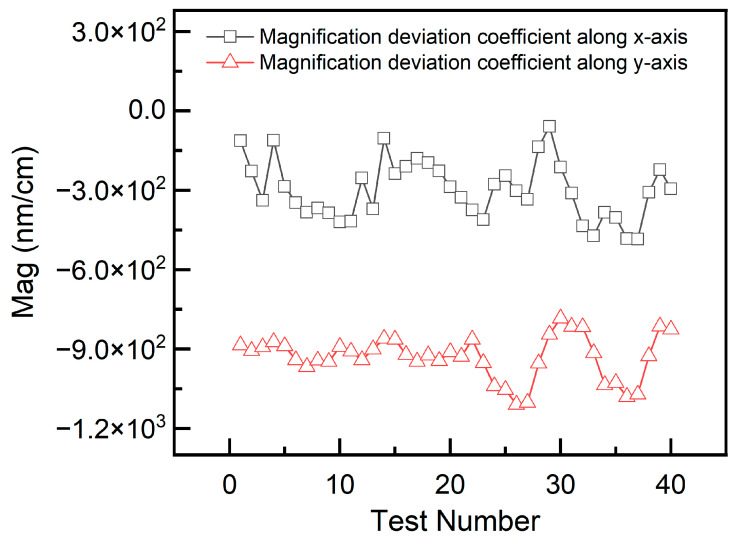
Repeatability test result curves of the magnification factor.

**Figure 15:**
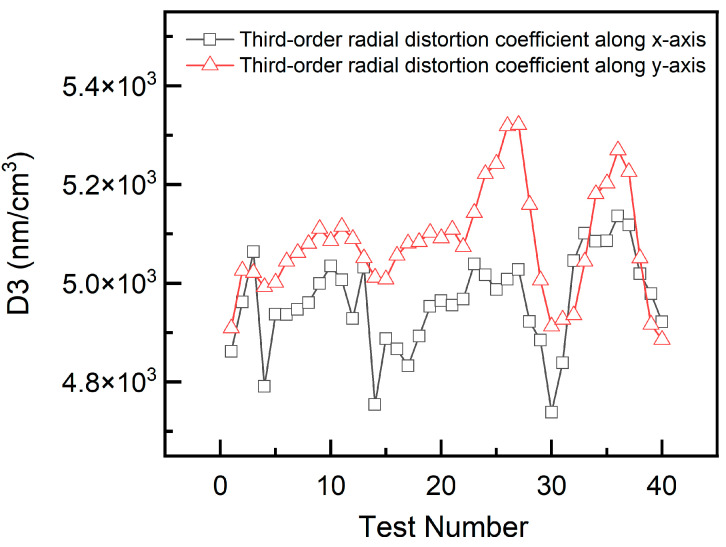
Repeatability test results for third-order distortion coefficients.

The repeatability of the method and apparatus was assessed using multiple measurements. In these experiments, a rectangular fringe target was placed at a height of 0 mm in the object plane of the lens. Phase shifting was repeatedly performed, and the magnification factor and third-order distortion coefficient were calculated. The results are presented in Figure 14 and Figure 15. Based on the 1-sigma principle, the measurement repeatability values for the magnification factor were determined to be ±108 and ±81 nm/cm in the *x*- and *y*-directions, respectively. For the third-order distortion coefficient, the measurement accuracies were ±93.6 and ±108 nm/cm^3^ in the *x*- and *y*-directions, respectively.

A comparative evaluation between the Ronchi test and the proposed Nyquist sampling method was conducted at the nominal object-plane position (*z* = 0). The quantitative measurement results are summarized in Table 2. Notably, the values for both magnification (ΔM) and third-order distortion (D3) in Table 2 represent the arithmetic mean of multiple independent measurements, ensuring statistical reliability.

Evidently, the results obtained using the Nyquist sampling method are in excellent agreement with the Ronchi reference data. The minimal deviations observed (which fall within the uncertainty budget of the Ronchi method itself) explicitly demonstrate the high measurement accuracy and reliability of the proposed Nyquist sampling method.

The test results are influenced by systematic and random errors. Systematic errors do not vary with environmental changes and represent a fixed component of the test results. Conversely, random errors, influenced by factors such as temperature fluctuations and phase-shift stability, are the primary contributors to the repeatability precision of the measurements. In this experiment, systematic errors primarily included target fabrication errors, temperature deviation between the image sensor and environment, and errors due to higher-order harmonic effects. The target fabrication error was 50 nm, corresponding to a position deviation of ±25 nm; the temperature of the image sensor was approximately 24.3 °C, with a 2 °C deviation from the ambient temperature of 22.2 °C, resulting in a linear expansion of approximately 83.2 nm over the 16 mm detection range. Higher-order harmonic errors were evaluated using Equation (30): with an MTF of 0.5 at the fundamental frequency and with the lens nearly attenuating the third harmonic (assessed as 0.01), the calculated error was approximately 9 nm. These systematic errors are the main causes of the deviation of results from those of the Ronchi test. However, as inherent properties, they do not affect the repeatability precision of the measurements. Random errors primarily included the effects of temperature variations on the image sensor, target, and lens mounting structure; they also included algorithmic errors and the impact of phase-shift position deviations from the piezoelectric translation stage. In this experiment, during the 40-test sequence, the ambient temperature variation was less than 0.1 °C, and the temperature fluctuations of the target, image sensor, and lens mounting structure were below 0.04 °C. The corresponding linear expansions for the image sensor and target over the 16 mm range were 1.7 and 0.35 nm, respectively, which mainly affected the magnification factor. The lens support structure comprised a cage system, with an evaluated short-term relative displacement of less than 10 nm. This displacement primarily influenced the relative positions of the lens, target, and image sensor, manifesting as $T_{x}$ and $T_{y}$ offsets in the distortion formula, while negligibly influencing the magnification and third-order distortion. The phase-shift position deviation of the piezoelectric stage was 30 nm, with a simulated error contribution of 5 nm, which affected both magnification and third-order distortion. The repetitive positioning accuracy of the target rotation mechanism was 20 arcseconds, which mainly influenced the rotation coefficient without affecting magnification or third-order distortion. The error introduced by the 4 × 4 binning process was 4 nm. By effectively controlling the temperature variations and phase-shift precision, each random error component was controlled to the nanometer level, ensuring the repeatability precision of the test results.

## 6. Conclusions

In this study, a detector was employed to capture images of rectangular fringe patterns at the Nyquist frequency. The distortion distribution of the tested lens was determined by analyzing the Moiré-fringe characteristics and applying the phase-shifting method. By integrating the distortion formula, the relationship between magnification and third-order distortion across different conjugate imaging planes was calculated. The experimental results show that the image-plane distortion distribution exhibited a consistent linear trend when the object-plane position varied within a limited spatial range. Repeatability tests confirmed that this method achieved a magnification deviation factor repeatability accuracy of approximately ±108 nm/cm and third-order distortion-measurement accuracy of approximately ±108 nm/cm^3^, thereby enabling a high-precision distortion evaluation for conventional industrial imaging lenses. Furthermore, enhancing the alignment accuracy of the testing system can improve overall measurement precision. For lenses with higher magnification, alternative target sources such as high-resolution displays capable of generating programmable fringe patterns offer feasible testing options.

## Figures and Tables

**Table 1:** Measurement uncertainty budget for the Ronchi test setup.

Uncertainty Source	Uncertainty Type	Probability Distribution	Standard Uncertainty (nm)
Repeatability	Type A	Normal	2.9
Vibration Influence	Type B	Normal	6.7
Quantization Error	Type B	Rectangular	4.4
Phase-Shifting Error	Type B	Rectangular	3.3
Light Source Stability	Type B	Rectangular	2.5
Ronchi Mark Position Deviation	Type B	Rectangular	14.4
Other Residuals	Type B	-	2.5
Combined Standard Uncertainty (u_c_)	-	-	17.4
Expanded Uncertainty (U, k = 2)	-	-	34.8

**Table 2:** Comparison of results obtained from Nyquist sampling and Ronchi tests.

Distortion Parameters	Nyquist Sampling Results	Ronchi Test Results
$\Delta M_{x}$ (nm/cm)	−299	−325
$\Delta M_{y}$ (nm/cm)	−931	−891
$D_{3x}$ (nm/cm^3^)	4960	4912
$D_{3y}$ (nm/cm^3^)	5080	5147

## Data Availability

The raw data supporting the conclusions of this article will be made available by the authors on request.

## Abbreviations

The following abbreviations are used in this manuscript:

MTF	Modulation transfer function
OTF	Optical transfer function
CTF	Contrast transfer function
PTF	Phase transfer function
LED	Light-emitting diode
